# A Rare Presentation of Epstein-Barr Virus-induced Hepatitis With an Interesting Twist: A Case Report Involving a Clinical Pearl

**DOI:** 10.7759/cureus.2808

**Published:** 2018-06-14

**Authors:** Simcha Weissman, Gordon Auyeung, Ali Atoot, Adam Atoot

**Affiliations:** 1 Medical Student, Touro College of Osteopathic Medicine, New York, USA; 2 Family Medicine, Palisades Medical Center, New Jersey, USA; 3 St George University Medical Center, St George, Grenada, GRD; 4 Internal Medicine, Palisades Medical Center, New Jersey, USA

**Keywords:** epstein-barr virus (ebv), hepatitis a, liver enzymes, infectious mononucleosis

## Abstract

An Epstein-Barr Virus (EBV) infection generally presents with the classic infectious mononucleosis symptomatology—sore throat, lymph node enlargement, and fatigue. However, there are cases in which induced hepatitis does occur. The liver enzymes leaked during the acute damage would be apparent in the serum as an elevation of transaminases and alkaline phosphatase. These elevations are almost always mild in nature. In addition, the associated presenting symptomatology is a generalized weakness, lethargy, and fatigue. Aside from the marked elevation of liver enzymes, what makes our case a rarity is the fact that the chief complaint was polydipsia and polyuria. Furthermore, an initial negative hepatitis A panel was found to be positive weeks later. The insight offered by this case is a true clinical pearl—namely, that the seroconversion of hepatitis A takes several weeks to occur.

## Introduction

Epstein-Barr Virus (EBV) infection—responsible for the notorious clinical syndrome of infectious mononucleosis—is almost always a self-limited disease characterized by sore throat, fatigue, and lymphadenopathy. Although liver involvement is nearly universal in a patient with Epstein-Barr Virus, it is usually mild and undetected clinically, and its resolution is spontaneous in nature [1]. Typically, EBV-induced hepatitis is characterized by mild, or perhaps even moderate, elevations of transaminases and alkaline phosphatase [2]. We present a rare case of EBV-induced hepatitis concomitantly infected with viral hepatitis A, including several valuable clinical teaching points.

## Case presentation

Our patient is a 44-year-old Caucasian female who presented to her primary care physician with a chief complaint of incredible thirst and increased frequent urination. The patient stated she returned two days ago from a vacation to Aruba where she spent the past week. On the last day of the trip, she began feeling these symptoms. Aside from the extreme polydipsia, she further admits to nausea, headaches, and malaise. She denied any fever, chills, weight loss, rash, and abdominal or joint pain. No other family member was sick. However, she admits to eating out in many restaurants off the resort, where there may have been seafood served. She has no other pertinent medical, surgical, or family history. She is not taking any medications, but is allergic to sulfa drugs. She denies the use of drugs, alcohol, or tobacco. She states she is sexually active, as she is in a monogamous relationship with her husband. She traveled alone and no other contacts were reported to be ill. On physical exam, she was afebrile and her vital signs were within normal limits. She appeared well-nourished, alert, and oriented, and her mucous membranes were moist. Her cardiovascular, pulmonary, abdominal, and genitourinary system exam findings were benign.

Laboratory studies undertaken by her primary care physician (PCP) revealed a serum alkaline phosphatase of 577 U/L (normal 39-117 U/L), aspartate aminotransferase (AST) of 376 U/L (normal 0-40 U/L), alanine aminotransferase (ALT) of 474 U/L (normal 0-32 U/L), and a total bilirubin of 2.9 mg/dl (normal 0.0-1.2 mg/dl). Upon receiving these laboratory results (of highly elevated LFTs), the patient was advised by her PCP to present to the emergency department (ED) for further workup. Upon admission to the hospital, she admitted to a history of eating a good amount of seafood—including shrimp and grouper—during her vacation to Aruba. At this time, she complained of epigastric abdominal pain and a continuation of her symptomatology. On physical exam, she was afebrile, had a BP of 122/91, a heart rate (HR) of 91, and a respiratory rate (RR) of 19, with a peripheral capillary oxygen saturation (SpO2) of 98%. She appeared well-nourished and was in no acute distress. On abdominal exam, she was tender to palpation in her epigastric region with a soft, non-distended abdomen and mild to moderate splenomegaly. Cardiovascular, pulmonary, and genitourinary system exam findings were benign.

Upon admission, laboratory studies revealed a white blood cell (WBC) of 11.3 (normal 4.0-11.0 x10(3)/mcl), serum alkaline phosphatase of 583 U/L (normal 39-117 U/L), aspartate aminotransferase (AST) of 356 U/L (normal 0-40 U/L), alanine aminotransferase (ALT) of 490 U/L (normal 0-32 U/L), and a total bilirubin of 3.1 mg/dl (normal 0.0-1.2 mg/dl). The urine pregnancy test came back negative. Right upper quadrant ultrasound (RUQ U/S) showed no dilatation of the common bile duct (CBD), the absence of a sonographic Murphy sign, mild pericholecystic fluid, with a 9 mm thickening of the gallbladder wall, suspicious for acute cholecystitis. A hepatobiliary iminodiacetic acid (HIDA) scan and computed tomography (CT) abdomen/pelvis showed benign findings, hence ruling out pathological biliary or gallbladder involvement.

Influenza A and B came back negative. Antinuclear antibody (ANA) and human immunodeficiency virus (HIV) antigene-antibody (Ag/Ab) were negative and nonreactive, respectively. The hepatitis panel drawn was negative for Hep A immunoglobulin M (IgM) (0.38, normal 0.00-0.79 s/c), HBS AG (0.14, normal 0.00-0.99 s/c), Hep B Core IgM (0.07, normal 0.00-0.99 s/c), and Hep C AB (0.06, normal 0.00-0.99 s/c). The patient was given fluids and her liver function tests (LFTs) began to trend downward over the next 24 hours, whereupon she was discharged with a diagnosis of “elevated LFTs.” She then presented to her primary-care physician (PCP) for post-discharge follow-up, whereupon a more aggressive workup was done. A heterophil antibody test was negative and, thus, EBV antibody titers were drawn. They were consistent with acute primary EBV Infection.

EBV viral capsid antigen (VCA) IgM was >160 (negative <35.9 U/mL and unequivocal 36.0-43.9), EBV EA IgG was 51.1 (negative <0.9 and unequivocal 9.0-10.9), EBV VCA IgG was 37.7 (negative <18.0 and unequivocal 18.0-21.9), EBV NA IgG was <18.0 (negative <18.0). In addition, smooth muscle antibody—actin Ab was 20 units (negative 0-19, weak positive 20-30, moderate to strong positive >30). Thus, hinting toward a hepatitis co-infection, as smooth muscle antibodies are found in 52%-85% of hepatitis patients. At this time, nearly 6 weeks from the beginning of her vacation, a hepatitis A panel was redrawn, as it was still high on the differential regardless of the initial negative results. Sure enough, this time around, the total hep A antibody was positive. The patient was encouraged to increase fluid intake with adequate electrolytes. Additionally, the patient was educated further on refraining from participating in contact sports until her physical exam returned to normal and there was no splenomegaly present. At her next PCP visit two weeks later, her LFTs were practically back to normal and her symptomatology had significantly decreased.

## Discussion

EBV is a ubiquitous human herpesvirus mostly known for its presentation as infectious mononucleosis. Although not common, other presentations can include extreme thirst or a generalized weakness and fatigue, as seen in our case. EBV-induced hepatitis has been documented previously, as elevated liver enzymes are quite common but in mild to moderate numbers. Higher numbers of liver enzyme elevation should prompt a more thorough workup and possibly suggest a co-infection. Understanding blood antigen/antibody serology is essential in making a proper diagnosis, given the correct etiology. Acute primary infection would show a positive EBV-IgM and VCA-IgG, with a positive or negative EA (D)-IgG and a negative EBNA-IgG. Convalescence/past infection would show a negative EBV-IgM, a positive or negative EA (D)-IgG, with a negative VCA-IgG and EBNA-IgG. Reactivated infection would show a positive or negative EBV-IgM with a negative EA (D)-IgG, VCA-IgG, and EBNA-IgG.

Treatment in these patients is supportive—namely to remain hydrated with an adequate electrolyte balance. Precautions to avoid contact sports are also important to emphasize, as there is a 0.1%-0.5 % risk of splenic rupture, which has been shown to be fatal [3]. Hepatitis A is a non-enveloped (naked) single-stranded RNA virus that is part of the picornavirus family. HAV is acquired through fecal-oral transmission and its replication occurs in the liver, only naturally elevating enzymes. Thus, persons who drank contaminated water or even ate seafood in an endemic region are at highest risk for the disease. Hepatitis A cannot be distinguished from other types of viral hepatitis solely on the basis of clinical symptoms, thus serologic testing is required to confirm the diagnosis. Virtually, all persons with acute hepatitis A have detectable IgM anti-HAV in the serum. In fact, acute HAV infection is confirmed during the acute or early convalescent phase of infection by the presence of IgM anti-HAV in serum. Important to note is that the incubation period for seroconversion to occur is roughly 28 days (range: 15-50 days) [4]. Thus, when clinical symptoms persist, appropriate measures should be taken to retest for HAV once the allotted time has passed and seroconversion has occurred indefinitely [5]. IgG anti-HAV appears in the convalescent phase of infection and remains present in serum for the entire lifetime of the person, thus ensuring protection against disease.

Unfortunately, there is no definitive treatment for the hepatitis A virus. The management of an HAV infection is namely supportive in nature, with electrolyte replacement vital for adequate hydration.

## Conclusions

The content of this manuscript serves to enlighten primary care and emergency medicine physicians about the odd way in which a case like this may present, as well as to aid and ensure that it is worked up appropriately in terms of etiology. One should now be aware that EBV can, in fact, induce hepatitis with even marked elevations in liver enzymes. Additionally, one should not rule out a hepatitis A coinfection without allowing for the allotted time in which seroconversion can occur.
